# Artificial Intelligence in Predicting Cardiac Arrest: Scoping Review

**DOI:** 10.2196/30798

**Published:** 2021-12-17

**Authors:** Asma Alamgir, Osama Mousa, Zubair Shah

**Affiliations:** 1 College of Science and Engineering Hamad Bin Khalifa University Qatar Foundation Doha Qatar; 2 Centre for Health Informatics Australian Institute of Health Innovation Macquarie University Sydney Australia

**Keywords:** artificial intelligence, machine learning, deep learning, cardiac arrest, predict

## Abstract

**Background:**

Cardiac arrest is a life-threatening cessation of activity in the heart. Early prediction of cardiac arrest is important, as it allows for the necessary measures to be taken to prevent or intervene during the onset. Artificial intelligence (AI) technologies and big data have been increasingly used to enhance the ability to predict and prepare for the patients at risk.

**Objective:**

This study aims to explore the use of AI technology in predicting cardiac arrest as reported in the literature.

**Methods:**

A scoping review was conducted in line with the guidelines of the PRISMA (Preferred Reporting Items for Systematic Reviews and Meta-Analyses) extension for scoping reviews. Scopus, ScienceDirect, Embase, the Institute of Electrical and Electronics Engineers, and Google Scholar were searched to identify relevant studies. Backward reference list checks of the included studies were also conducted. Study selection and data extraction were independently conducted by 2 reviewers. Data extracted from the included studies were synthesized narratively.

**Results:**

Out of 697 citations retrieved, 41 studies were included in the review, and 6 were added after backward citation checking. The included studies reported the use of AI in the prediction of cardiac arrest. Of the 47 studies, we were able to classify the approaches taken by the studies into 3 different categories: 26 (55%) studies predicted cardiac arrest by analyzing specific parameters or variables of the patients, whereas 16 (34%) studies developed an AI-based warning system. The remaining 11% (5/47) of studies focused on distinguishing patients at high risk of cardiac arrest from patients who were not at risk. Two studies focused on the pediatric population, and the rest focused on adults (45/47, 96%). Most of the studies used data sets with a size of <10,000 samples (32/47, 68%). Machine learning models were the most prominent branch of AI used in the prediction of cardiac arrest in the studies (38/47, 81%), and the most used algorithm was the neural network (23/47, 49%). K-fold cross-validation was the most used algorithm evaluation tool reported in the studies (24/47, 51%).

**Conclusions:**

AI is extensively used to predict cardiac arrest in different patient settings. Technology is expected to play an integral role in improving cardiac medicine. There is a need for more reviews to learn the obstacles to the implementation of AI technologies in clinical settings. Moreover, research focusing on how to best provide clinicians with support to understand, adapt, and implement this technology in their practice is also necessary.

## Introduction

### Background

Cardiac arrest, also known as sudden cardiac death, is the cessation of the ability of the heart to pump blood. This acute cessation requires immediate intervention, as vital organs, such as the brain and the heart itself, are deprived of blood flow. A delay in intervention can lead to lifelong complications and even death. The global rate of mortality after cardiac arrest is significantly high—78% of out-of-hospital cardiac arrest (OHCA) cases die before they reach the hospital [1]. For those who do receive advanced care, the survival rate remains low. The survival rate for OHCA from the time of cardiac arrest to the time of discharge ranges from 2% to 11% worldwide [2]. The number of cardiac arrest deaths that occur within an in-hospital setting is also significant. In the United States alone, over 290,000 in-hospital cardiac arrests occur annually, with survival rates varying from as low as 0% to 36.2%, out of which a small percentage have favorable neurological prognoses [3].

Artificial intelligence (AI) is reforming health care every day. AI technologies have the perfect platform to thrive and mature with the growing adoption of electronic health records, development in computational power, continuous monitoring systems, and availability of big data [4]. It has become an important clinical decision-making tool that allows for personalized diagnoses, solutions, prognoses, and predictions of future health outcomes, guiding clinicians and other stakeholders in doing what is best for their patients [4]. AI technology is also rapidly progressing in cardiology, like in any other field of medicine [5]. AI-guided diagnosis and therapy selection have allowed for advancement in research, clinical practice, and population health in cardiovascular medicine [6]. Machine learning (ML) models have also been shown to outperform traditional statistical models in detecting sex differences in cardiovascular disease, further enhancing individualized medicine [7]. AI also plays a major role in improving care for cardiac arrest. AI technologies are being used to prevent cardiac arrest through early identification of risk factors [8], early detection [9], improved management (eg, effective cardiopulmonary resuscitation) [10], and prognosis determination for patients post cardiac arrest [11]. A large part of cardiac arrest research is the prediction of cardiac arrest before its occurrence, as it gives clinicians time to prepare and achieve better patient outcomes.

Thus, what are AI technologies and their counterparts in this context? AI refers to the field of science revolving around building computational systems and algorithms that facilitate the ability of a machine to mimic human behavior to learn and find solutions to tasks autonomously [4,12]. ML is a subset of AI. ML algorithms focus on building smart solutions after learning from patterns and experiences provided by a structured sample of training data [12]. Deep learning (DL) is a class of ML. It consists of a complex, interconnected, multilayered neural network, resembling a human brain. The aim of DL is to learn and understand patterns from a large amount of unstructured data [5]. In short, the more information it is fed, the more accurate the outcome.

The ability of AI technologies to process and evaluate patient data to generate predictions is important to support clinicians in making critical decisions, provide effective management, and, ultimately, improve patient outcomes in cardiac arrest cases [13]. Therefore, we believe it is crucial to explore the use of AI technology in predicting cardiac arrest and report our findings to help clinicians and researchers.

### Research Problem and Aim

Numerous studies have proposed the use of AI in cardiac care, especially the use of AI in the prediction of cardiac arrest. However, there is a lack of consolidating existing evidence that describes the features of AI technologies, data sets, and data sources currently being used. It is essential to summarize recent findings that allow health care providers and researchers to implement appropriate guidelines, as well as to identify research gaps in the current literature. We encountered one review that examined the use of AI in the prediction of cardiac arrest [14]. However, the review was conducted in 2018 and did not include a large influx of studies in the past 2 years. Therefore, it is necessary to conduct a scoping review that focuses on various types of AI technologies currently being used in different settings to predict cardiac arrest.

This scoping review aims to explore the use and features of AI technologies applied to the prediction of cardiac arrest as reported in the literature. The results of our review will be a useful reference for health care professionals, researchers, and others involved in patient care to understand the application of AI and leverage it for the benefit of the community.

## Methods

The scoping review was conducted by AA and OM to address this objective. The guidelines of the PRISMA (Preferred Reporting Items for Systematic Reviews and Meta-Analyses) extension for scoping reviews [15] were followed to help conduct a transparent review.

### Search Strategy

Five bibliographic databases were searched for this study: Scopus, ScienceDirect, Embase, the Institute of Electrical and Electronics Engineers, and Google Scholar. The databases were searched using search terms related to the target technology, population, and outcomes of interest. Search terms for our population included *Cardiac Arrest* OR *Heart Arrest* OR *Sudden Cardiac Death* OR *asystole* OR *cardiopulmonary arrest* and, for our intervention, *Artificial Intelligence* OR *Deep Learning* OR *Machine Learning* OR *Natural Language Processing* OR *Neural network* OR *Supervised learning* OR *Unsupervised learning* OR *Data mining*. Outcome- or purpose-related search terms included Detect* OR Predict* OR Anticipat* OR Diagnos*. The search query used for each database is presented in Multimedia Appendix 1.

For ScienceDirect and Google Scholar, only the first 100 and 50 results, respectively, were considered. This is because the reviewers found that the results became less relevant to the topic of interest and applicability after the mentioned number of citations. In addition to searching the databases, a backward reference list screening of the included studies was also carried out to identify additional relevant studies. The search was conducted between March 15 and 20, 2021.

### Eligibility Criteria

AI technologies implemented to predict cardiac arrest were included, with no restrictions on age, gender, geography, and type of AI technology used. Studies that focused primarily on predicting cardiac arrest were included. In contrast, studies dedicated to other aspects or contributing factors of cardiac arrest, such as arrhythmia and other cardiac diseases, were excluded. The review included peer-reviewed articles, preprints, articles in press, conference proceedings, theses, and dissertations written in English. Reviews, conference abstracts, study protocols, and proposals were excluded. No restrictions were imposed on the study design, study setting, country of publication, and publication year during the search query. However, only studies published between 2013 and 2021 were included in the review. The period between 2013 and 2016 constitutes a time when AI technologies saw a rapid increase of 175% in application [16]; therefore, the reviewers considered it to be a reasonable time period to include. The study eligibility criteria are summarized in Textbox 1.

**Inclusion and exclusion criteria.**

**Inclusion criteria**

- Studies that focused on the use of artificial intelligence (AI) technologies in cardiac arrest prediction for the benefit of the human population
- Studies published from 2013 to 2021
- Peer-reviewed articles, articles in press, theses, dissertations, and conference proceedings
- Primary studies

**Exclusion criteria**

- Articles that did not address the use of AI in cardiac arrest prediction
- Reviews, conference abstracts or proposals, letters, news, books, and protocols
- Published in a language other than English

### Study Selection

The studies retrieved from the databases were first imported to Rayyan (Rayyan System Inc) [17], a collaborative research tool, to undergo 3 phases of the filtering process. This ensured that the articles we included in the review were relevant to our study objective. The 3 phases of the filtering process were as follows: (1) identification phase, where citations were identified after applying the search terms to the databases and duplicates were removed; (2) screening phase, where titles and abstracts were screened to remove articles that did not match our inclusion criteria; and (3) eligibility phase, where the full texts of the articles were read to determine their applicability on the basis of the inclusion criteria. The 2 reviewers conducted all 3 phases independently, facilitated by the Rayyan application. In case of conflict, a discussion was held to reach a consensus.

### Data Extraction and Data Synthesis

To conduct a reliable and consistent extraction of data from the included studies, a data extraction form was used (Multimedia Appendix 2). The 2 reviewers independently extracted data related to the characteristics of the included studies, AI technology, and data sets. The extracted information was recorded on a shared Microsoft Excel sheet for easy data management. Similar to the study selection, any conflict between the 2 reviewers was resolved through discussions to reach a consensus.

A narrative synthesis of the extracted data was performed. The findings from the included studies were classified and described in terms of their purpose, AI branch, algorithm, and platform used to implement the algorithm. The data sets used for the development and validation of the technology were considered and described. The data sources, size of the data set. validation type, and proportion of training, validation, and test data sets were included when available. An sheet Excel (Multimedia Appendix 3) was used to record the extracted data to facilitate data synthesis.

## Results

### Search Findings

**Figure 1:**
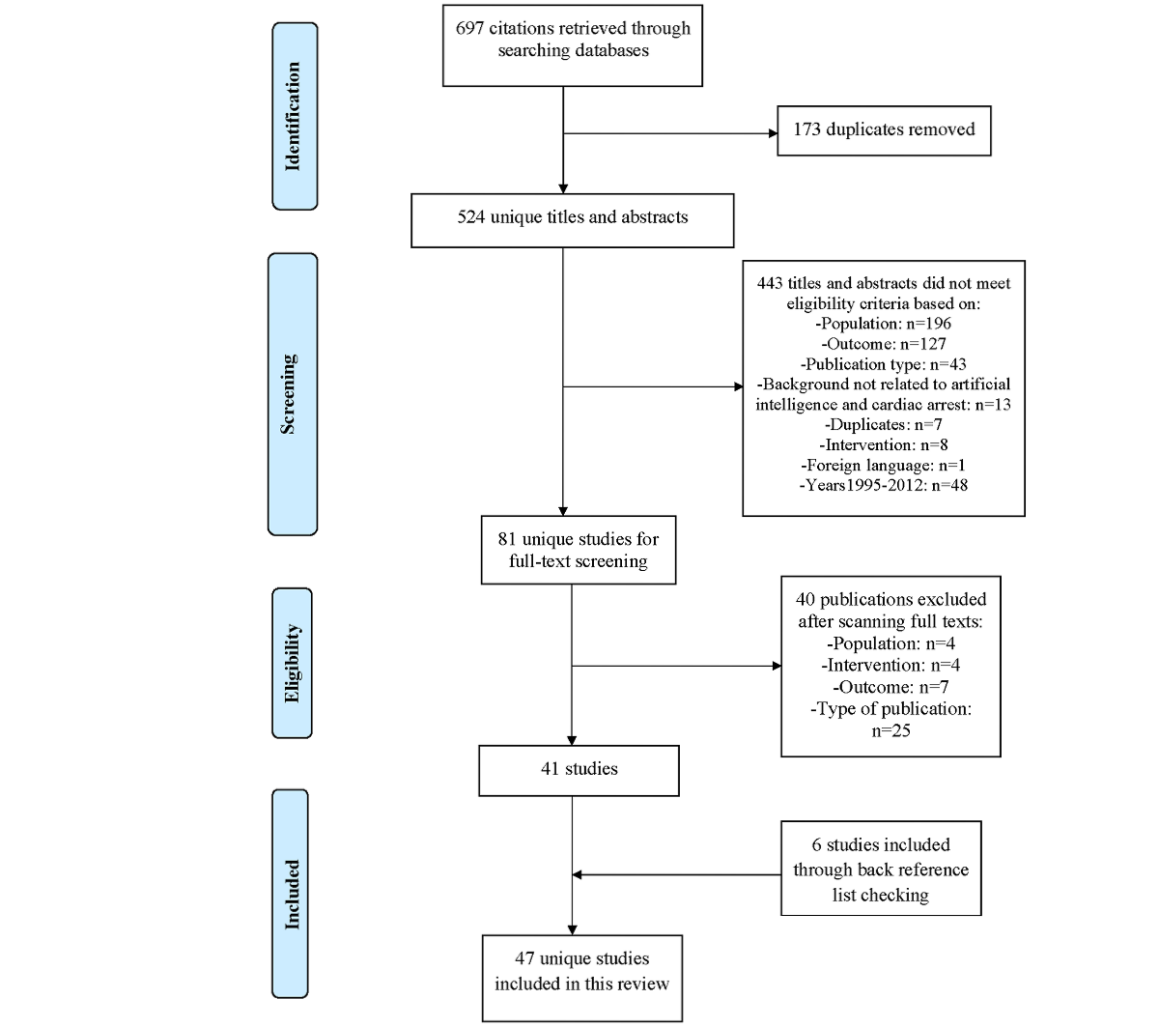
Flowchart of the study selection process.

As shown in Figure 1, 697 studies were retrieved from our search, of which 173 (24.8%) duplicates were removed. A total of 524 underwent title and abstract screening, of which 443 (84.5%) studies were excluded. The reasons for exclusion are shown in Figure 1. In total, 81 unique studies underwent full-text screening to evaluate eligibility, of which 41 (51%) studies met the inclusion criteria and were included in the review. Six additional studies were identified and added by checking the reference lists of those 41 studies. Overall, 47 studies were included in the review.

### Characteristics of the Included Studies

Of the 47 studies included, 46 (98%) were published in peer-reviewed journals, whereas 1 (2%) was still in press. Approximately 81% (38/47) of the studies were research articles, whereas the rest were conference proceedings (9/47, 19%). Only 2 studies from 2013 were included, whereas most of the studies were from 2020 (12/47, 26%). The other included studies were conducted in 2014 (4/47, 9%), 2015 (5/47, 11%), 2016 (3/47, 6%), 2017 (3/47, 6%), 2018 (5/47, 11%), 2019 (9/47, 19%), and 2021 (4/47, 9%). The included studies were conducted in 15 countries, and most of the studies were published in India and the United States (9/47, 19%). Table 1 shows the characteristics of the studies included in our review. Multimedia Appendix 3 demonstrates the attributes of each study.

**Table 1:** Characteristics of the included studies (N=47).

Characteristic			Studies, n (%)
**Paper status**			
	Published	46 (98)	
	In press	1 (2)	
**Publication type**			
	Conference proceeding	9 (19)	
	Research article	38 (81)	
**Country**			
	Australia	1 (2)	
	China	3 (6)	
	Greece	1 (2)	
	India	9 (19)	
	Iran	5 (11)	
	Japan	1 (2)	
	Malaysia	4 (9)	
	Poland	1 (2)	
	Portugal	1 (2)	
	Singapore	1 (2)	
	South Korea	7 (15)	
	Spain	1 (2)	
	Taiwan	2 (4)	
	United Kingdom	1 (2)	
	United States	9 (19)	
**Year published**			
	2013	2 (4)	
	2014	4 (9)	
	2015	5 (11)	
	2016	3 (6)	
	2017	3 (6)	
	2018	5 (11)	
	2019	9 (19)	
	2020	12 (26)	
	2021	4 (9)	

### AI Characteristics in the Included Studies

#### Use of AI in Predicting Cardiac Arrest

The approaches taken by the included studies to predict cardiac arrest using AI technologies were divided into 3 categories: analysis of variables and parameters, development of an early warning system or prediction model, and stratification of patients at a high risk of cardiac arrest.

#### Analysis of Variables and Parameters

The studies in this category focused on analyzing one or more patient parameters to determine their impact on the efficiency of improving the prediction of cardiac arrest in combination with AI algorithms. We observed 26 studies that fit into this category [14,18-42]. Of these 26 studies, 11 (42%) used ML models [14,18,19,23,25,27,30,32,34-36] and 3 (12%) used DL algorithms [20,31,38]. We observed that 12 studies incorporated both ML and DL models to analyze and validate different parameters [21,22,24,26,28,29,33,37,39-42].

Random forest (RF) [14,21,23,28-30,32,35-37,39-41] and support vector machine (SVM) [18,22,24,26,28,34,40-42] were the most used ML models observed in these studies, followed by decision tree (DT) [22,29,30,40-42], logistic regression (LR) [28-30,40], Naive Bayes [19,28,29,41], gradient boosting [27,28], extreme gradient boosting [27,29], LogitBoost [21], AdaBoost [29], TreeBagger [34], and sequential feature selection [24]. The most used DL-based algorithm in the studies was k-nearest neighbors (KNN) [20,22,26,29,33,41,42]. The probabilistic neural network [24,31,42], artificial neural network [29,40], multilayer perceptron [21,33], long short-term memory [39], convolutional neural network [37], and enhanced probabilistic neural network [31] were also algorithms used in DL model studies. Furthermore, 2 studies did not specify the algorithm used [25,38].

The parameters analyzed and validated in the included studies were diverse. The majority of the studies focused on using various characteristics from patient electrocardiogram readings [14,18,20-22,24,26,27,30-34,36-38,40-42], especially heart rate variability (HRV) [14,21,22,26,30,32,34,36-38,42]. HRV is the variation in time between each heartbeat that can be tracked on an electrocardiogram [43]. This noninvasive assessment tool provides important information about the autonomic nervous system, allowing clinicians to determine current and impending cardiac disease [44]. Its usefulness in determining cardiac-related prognosis is also well-documented in the literature [45,46]. In the included studies, HRV appeared to improve prediction outcomes in the studies that integrated it into the data set. All studies using HRV reported higher performance in terms of accuracy and other outcome indicators. Other unique parameters, such as genetic data [20], smoking habit [29], nursing documentation [25], and dialysis status [23,28], were also used to evaluate their effect on the performance of the AI technology to predict cardiac arrest. Accuracy [18,19,21,22,29,31,33,34,36,37,40,41] and sensitivity [14,22,26-28,30,32-34,41,42] were the most used measures of outcome in this category.

### Development of an Early Warning System Using AI

In 16 studies [47-62], the focus of AI technologies was to develop an early warning system alerting health care professionals when patients were at risk of going into cardiac arrest in the future. To develop a warning model, most studies used ML model algorithms [49-51,53,54,59,61], whereas 5 only used DL-based algorithms [47,48,56,60,62]. Four studies used both ML- and DL-based algorithms [52,55,57,58], comparing them with each other to observe which yielded the best outcome. The ML algorithms used in these studies included LR [50,52,55,58], SVM [50-52,58], DT [52,53,57,59], RF [55,57,58], Naive Bayes [57,58], gradient boosting [58], Bayesian networks [49], AdaBoost [57], transfer learning [54], and multichannel Hidden Markov Model [61]. The KNN [52,58], artificial neural network [48,58,61], long short-term memory [47,56,59], and recurrent neural network [47,55,56,62] algorithms were used in the studies to constitute a DL-based early warning system. A total of 10 of the studies compared their outcomes to existing or *traditional* early warning systems [47,48,50,52,53,56-58,60,62]. The studies compared their models to scoring systems such as the Modified Early Warning Score [48,50,52,53,56,58,60,62], Early Warning Score [57], National Early Warning Score [60], and Pediatric Early Warning Score [47]. Only 1 study showed similar outcomes when using an AI model compared with a traditional warning system [53], whereas, in other studies, the AI-based model outperformed the system it was compared with. For example, deep early warning systems detected 50%-78% more cardiac arrests compared with the Modified Early Warning Score [56,62]. Moreover, the prediction period of the algorithms was reported to range from 30 minutes to as early as 24 hours before the onset of cardiac arrest [50,53,57,58,62].

Three of the most used outcome measures in this category included the area under the receiver operating characteristic curve [47,48,51,53,55-57,60,62], sensitivity [49,52,58,60,62], and accuracy [51,54,58-60].

### Stratification of High-risk Patients

In 5 studies [63-67], AI technologies were used to distinguish patients who were at high risk of cardiac arrest from patients who were not at risk. Three studies highlighted HRV [63-65] as an important feature to distinguish high-risk patients.

ML was used in the majority of the studies [63,64,67], and only 1 study used a DL algorithm [66]. One study used both ML and DL models to stratify patients [65]. The ML algorithms used were SVM [63,64], linear discriminant analysis [64], DT [63], LR [67], RF [67], extreme gradient boosting [67], and fuzzy classifier [65]. The DL algorithms included KNN [65,66] and multilayer perceptron [66]. The outcome measures in the studies included accuracy [63-66], sensitivity, specificity [63-65], area under the receiver operating characteristic curve, and the precision-recall curve [66].

### Features of AI Techniques in the Studies

Most studies used traditional ML models and algorithms to predict cardiac arrest (38/47, 81%) whereas 55% (26/47) used DL techniques. We observed 15 types of AI classifiers used in the studies to predict cardiac arrest **(**Table 2**)**. A notable observation is that 6 models were commonly used; neural network–based models, which are a DL model, and RF, which is a traditional ML model, were used 20 and 18 times, respectively, making them the top 2 most used models found in the studies, followed by SVM (15/47, 32%), DT (12/47, 26%), LR (11/47, 23%), and KNN (10/47, 21%). Less common models, such as transfer learning, linear discriminant analysis, fuzzy classifier, multichannel Hidden Markov Model, LogitBoost, AdaBoost, Bayesian networks, Naive Bayes, and extreme gradient boosting, were used between 1 and 6 times in the studies. Two studies used wearable devices as the platform for their AI techniques [24,59], whereas the remaining studies used computers. Multimedia Appendix 3 presents the features of the AI techniques.

**Table 2:** Features of artificial intelligence (AI)–based techniques used for cardiac arrest prediction (N=47).

Feature			Study ID^a^		Studies, n (%)^b^
**AI model^c^**					
	Neural network	1, 3, 4, 6, 11, 13, 14, 15, 16, 19, 21, 25, 26, 28, 32, 34, 26, 38, 45, 46		20 (43)	
	Random forest	3, 6, 7, 8, 9, 10, 13, 14, 15, 17, 18, 19, 28, 20, 35, 37, 41, 45		18 (38)	
	Support vector machine	2, 5, 19, 20, 27, 30, 31, 32, 34, 38, 41, 42, 43, 45, 46		15 (32)	
	Decision tree	3, 5, 15, 16, 17, 18, 19, 20, 32, 34, 40, 42		12 (26)	
	Logistic regression	3, 6, 10, 15, 16, 18, 19, 30, 32, 45, 47		11 (23)	
	K-nearest neighbors	3, 20, 24, 32, 33, 34, 36, 42, 43, 46		10 (21)	
	Extreme gradient boosting	3, 10, 15, 16, 44, 45		6 (13)	
	Naive Bayes	16, 20, 22, 45		4 (9)	
	AdaBoost	15		1 (2)	
	Bayesian networks	29		1 (2)	
	LogitBoost	28		1 (2)	
	Multichannel Hidden Markov Model	23		1 (2)	
	Fuzzy classifier	33		1 (2)	
	Linear discriminant analysis	27		1 (2)	
	Transfer learning	47		1 (2)	
**Platform**					
	Computer	1-16, 18-37, 39-47		45 (96)	
	Wearable	17, 38		2 (4)	

^a^The order of the reviewed studies in this table follows the order shown in Multimedia Appendix 3.

^b^Two studies did not specify the artificial intelligence model used.

^c^The numbers do not add up as some studies used more than one artificial intelligence model or algorithm.

### Features of Data Sets Used for Development and Validation of AI Models

Clinical setting sources (such as hospital databases and medical centers) were the most commonly used data sources for the development and validation of AI models [14,25,27,28,31,32,34-36,38,39,47-53,55-57,60,62,67]. Public resources (eg, the MIT-BIH Arrhythmia and Normal Sinus Rhythm databases) [18-24,26,29,30,33,37,41,42,54,58,61,63-66] were the other sources of data for AI models.

Several types of data were retrieved from these sources. We grouped the types of data into 5 categories: clinical data, demographic data (eg, age, gender, and ethnicity), laboratory data (eg, blood samples), radiology data (eg, x-rays), and biological data (eg, genetic information). As shown in Table 3, 58% (34/47) of the studies used clinical data as the data type. Different variables fall under this category; Table 4 breaks down the type of clinical data observed in the studies. Demographic data were the second most used data type in predicting cardiac arrest (15/47, 26%), followed by laboratory data (8/47, 14%) and biological data (1/47, 2%).

**Table 3:** Data types.

Data type	Studies, n (%)
Clinical data	34 (72)
Demographic data	15 (32)
Laboratory data	8 (17)
Biological data	1 (2)

**Table 4:** Clinical data breakdown^a^.

Clinical data types	Studies, n (%)
Vital signs	23 (49)
ECG^b^ variables	18 (38)
Medical history	10 (21)
Chief complaint	3 (6)
Medication	3 (6)
Cardiopulmonary exercise testing	2 (4)
Diagnosis	2 (4)
Risk score	2 (4)
Renal status	2 (4)
Cardiopulmonary resuscitation information	1 (2)
Lifestyle	1 (2)
Nursing notes	1 (2)

^a^Several studies collected more than one clinical data type.

^b^ECG: echocardiogram.

For data set sizes, 42 (89%) out of 47 studies mentioned the size of the training data set used for the ML model. Of the 47 studies, 23 (49%) used data sets of less than 1000 samples, whereas 14 (30%) used data sets of between 1000 and 9999 samples. Moreover, 11% (5/47) of studies used more than 10,000 data samples. Various validation types for the AI models were reported in 41 studies. These validation methods were divided into 3 main categories: k-fold cross-validation, which was the most common validation technique used (24/47, 51%), followed by train-test split (11/47, 23%) and external validation (6/47, 13%). Table 5 provides a breakdown of the features of data used in the included studies.

**Table 5:** Features of the data used (N=47).

Feature		Studies, n (%)
**Data sources**		
	Public database	21 (45)
	Clinical setting	24 (51)
	Other	2 (4)
**Data set size^a^**		
	<1000	23 (49)
	1000-9999	14 (28)
	≥10,000	5 (11)
**Type of validation^b^**		
	K-fold cross-validation	24 (51)
	Train-test split	11 (23)
	External validation	6 (13)

^a^Data set size mentioned in 42 studies.

^b^Types of validation mentioned in only 41 studies.

## Discussion

### Principal Findings

In this review, we explored the use of AI in predicting cardiac arrest. From a total of 617 retrieved studies, 47 (7.6%) were included in this review. We found that the number of studies increased in the past 2 years (9 in 2019 and 11 in 2020), which is not surprising given that the use of AI technology in health care has been increasing. India and the United States (9/47, 19%) represent the countries that published the most studies related to AI in predicting cardiac arrest, with a total of 18. To explore the use of AI technology in predicting cardiac arrest, we divided our findings into 3 categories, each representing a classification of the reviewed studies from a different perspective. The first category focuses on the way AI technologies are used in predicting cardiac arrest and comprises 3 main subcategories: (1) stratification between patients with cardiac arrest and non-at-risk patients, in which the AI technology was trained using the history of patients who had cardiac arrest and classified patients with a high risk of cardiac arrest; (2) development of an early warning system using AI, in which AI technology was used to alert physicians 1 to 16 hours before cardiac arrest and its accuracy was compared with other existing traditional warning systems; and (3) analysis of different variables and parameters to observe the efficiency of prediction.

The second category identifies the features of the AI techniques as observed in the literature. Two AI branches were used, ML and DL, where ML was the most used branch in a total of 38 studies, and the most used model in this branch was RF (18/47, 38%). In contrast, DL was used 16 times, and the most used model were neural network–based models (20/47, 43%). Finally, the third category classifies the data and validation method used for the AI, where we expanded on the data sources, data types, and validation processes found in the literature for the AI techniques. A total of 42 out of the 47 studies mentioned the data set size used, the majority of the studies using data sets of less than 1000 samples (23/47, 49%). Most studies used k-fold cross-validation to test the AI models (24/47, 51%).

### The Implications for Practice and Research

This review highlighted the most common AI models used in predicting cardiac arrest and the different approaches used in predicting it. On the basis of our findings AI models can predict cardiac arrest using a variety of data types. In our review, ML techniques were used much more than DL techniques. One explanation for this is that the data used to train the AI model were mostly structured (eg, vital signs are recorded, and the threshold for the measurements of a normal human being is known and then compared with the vital signs of a patient who had cardiac arrest). Therefore, it is understandable that most researchers used ML techniques, because they were dealing with structured data. In contrast, DL works best with unstructured data, which was less commonly used in the articles reviewed. Another explanation is the size of the data sets used, as most studies used relatively small data sets to train DL models (eg, only 5 studies out of 47 used data sets of more than 10,000 samples). Finally, many studies explained the use of ML techniques such as DTs, LR, and RF, which consist of many DTs given that the main outcome is binary (at risk of cardiac arrest or not at risk of cardiac arrest). This explains the rapid use of these techniques in the reviewed studies.

Future research should explore ways to attain higher prediction accuracy in terms of the time before cardiac arrest may occur to the patient and the percentage of true positive and true negative (accurately predicting that the patient will experience cardiac arrest). Moreover, more research is required to address and investigate hyperparameter optimization, as it could lead to different performance results of ML models across the studies selected and influence which parameters are important for the prediction of cardiac arrest. Early prediction of cardiac arrest could be achieved through the correlation between the clinical data obtained and the demographic data of the patient. ML seems to be the best technique to be used because the data used is structured (eg, age, vital signs, and electrocardiogram variables). The earlier the prediction time, the higher the likelihood that the physicians can save the patients from sudden cardiac death. Furthermore, the potential to evaluate the effectiveness of less frequently used data types, such as laboratory and biological data, in predicting cardiac arrest should also be explored.

Only 5 studies reviewed used data sets of more than 10,000 samples, whereas most of the studies used data sets of less than 1000 samples. Future studies need to evaluate AI models using larger data sets to improve their effectiveness. In addition, comparing the prediction accuracy of AI techniques with each other is a good method of evaluation. However, AI techniques need to be compared with other techniques used to predict cardiac arrest.

Studies that did research in clinical settings limited the population to a specific hospital or country, which produced biased results that do not apply everywhere. Future studies should consider public databases that contain cases from different hospitals and countries.

Many studies explored the potential of AI in the prediction of arrhythmia and irregular heartbeat, and future studies should investigate the potential of the proposed models in the prediction of cardiac arrest. Finally, future research should explore the potential of physiological and psychological data in the prediction of cardiac arrest.

### Strengths

The review addressed the use of all types of AI technologies to predict cardiac arrest in all populations with no restrictions on paper status, study settings, and geographic location in a comprehensive manner. Moreover, an in-depth exploration was conducted on the features of AI technology and the data sets that were used to develop and validate these technologies.

Other reviews have explored the use of ML and DL in detecting arrhythmia [53,68] or the use of AI in cardiology in general [69-71] but have not gone into detail on how this technology can be used to predict cardiac arrest. A previous systematic review explored the use of ML in predicting cardiac arrest [72]; however, to the best of our knowledge, this is the first review to explore the different approaches to predicting cardiac arrest to fill the research gap with a better understanding of the prediction techniques rather than focusing on whether the model was able to predict only cardiac arrest. Moreover, this study did not focus on a specific AI branch (ML, DL, or natural language); rather, it focused on categorizing the AI techniques into branches to provide insight into the most common AI technique in every branch.

The studies included in the review comprised the latest publications, reducing the selection bias date. In addition to published research articles, conference proceedings were also included to maximize the extent of inclusion. This was also done by conducting a backward reference list check of the included studies. Furthermore, study selection and data extraction involved 2 reviewers independently overseeing the process, which ensured minimal selection bias.

### Limitations

This review did not include databases such as ACM and JSTOR, which limited our access to gray literature and other potentially relevant studies. This was because of the lack of access to some of the databases and others specialized in physiological or engineering studies rather than medical studies. Moreover, owing to practical constraints, only English-language studies were included in the review, excluding studies in other languages. Furthermore, our search query did not include MeSH (Medical Subject Headings) terms or algorithm-specific search terms, which might have hidden studies that would otherwise have been appropriate for our review.

### Conclusions

Our scoping review included 47 studies that focused on the use of AI technologies to predict cardiac arrest in all settings. With the big data available from patient monitoring systems and electronic health records, it is possible to delve deeper into making our approach to cardiac arrest reliable and more effective, increasing the rate of survival over time. Moreover, with the increasing adoption of wearable devices with sensors tracking various aspects of health and activity, there are opportunities for research to develop techniques to predict and alert patients at risk of OHCAs. Furthermore, clinicians need to be on board with the rapidly growing technology as, without them, we cannot move forward. Therefore, more research on AI paired with education initiatives within health care professionals needs to be considered.

## Appendix

Multimedia Appendix 1 Search strategy.

Multimedia Appendix 2 Data extraction form.

Multimedia Appendix 3 Characteristics of the included studies and features of artificial intelligence techniques.

## Abbreviations

AI: artificial intelligence
DL: deep learning
DT: decision tree
HRV: heart rate variability
KNN: k-nearest neighbors
LR: logistic regression
MeSH: Medical Subject Headings
ML: machine learning
OHCA: out-of-hospital cardiac arrest
PRISMA: Preferred Reporting Items for Systematic Reviews and Meta-Analyses
RF: random forest
SVM: support vector machine
