# Supplementary material for: Artificial Intelligence in Predicting Cardiac Arrest: Scoping Review
Source: JMIR Med Inform. 2021 Dec 17;9(12):e30798. doi: 10.2196/30798 (PMC8726033; doi:10.2196/30798)
Supplement: Multimedia Appendix 1 [file medinform_v9i12e30798_app1.docx]

### Multimedia Appendix

Multimedia Appendix 1

: Search Strategy

| Database | Search Query |
| --- | --- |
| ScienceDirect  First 100 chosen | (“Machine Learning” AND “Detect*” AND “Cardiac Arrest”) |
| Scopus  244 studies | ("Artificial Intelligence") OR ("Machine Learning") AND ("Detect*" OR “predict*”)AND (“sudden AND cardiac AND death”) OR (“heart AND arrest”) OR ("Cardiac Arrest") |
| Embase  269 studies | ('artificial intelligence'/exp OR 'artificial intelligence' OR 'deep learning'/exp OR 'deep learning' OR 'machine learning'/exp OR 'machine learning' OR 'natural language' OR 'neural network'/exp OR 'neural network' OR 'supervised learning'/exp OR 'supervised learning' OR 'unsupervised learning'/exp OR 'unsupervised learning') AND ('cardiac arrest'/exp OR 'cardiac arrest' OR 'heart arrest'/exp OR 'heart arrest' OR 'sudden cardiac death'/exp OR 'sudden cardiac death' OR 'asystole'/exp OR asystole OR 'cardiopulmonary arrest'/exp OR 'cardiopulmonary arrest') AND (detect* OR predict* OR diagnos* OR anticipat*) |
| IEEE  34 Studies | ("All Metadata":"Artificial Intelligence" OR "All Metadata":"Machine learning" OR "All Metadata":"Deep learning" OR "All Metadata":"Natural Language Process") AND ("All Metadata":"Predict*") AND ("All Metadata":"Cardiac arrest" OR "All Metadata":"Heart arrest" |
| Google Scholar  First 50 Studies | ("Artificial Intelligence" OR "Machine Learning" OR “Deep Learning”) AND ("Detect*" OR “predict*”) AND ("Cardiac Arrest" OR “Sudden cardiac death” OR “Heart arrest”) |
