# Supplementary material for: Artificial Intelligence in Predicting Cardiac Arrest: Scoping Review
Source: JMIR Med Inform. 2021 Dec 17;9(12):e30798. doi: 10.2196/30798 (PMC8726033; doi:10.2196/30798)
Supplement: Multimedia Appendix 2 [file medinform_v9i12e30798_app2.docx]

Multimedia Appendix 2

| Concept | Definition |
| --- | --- |
| Study Characteristics |  |
| Author | The first author of the study. |
| Year of Submission | The year in which the study was submitted. |
| Country of publication | The country where the study was published. |
| Paper status | The current status of the paper |
| AI technique characteristics |  |
| Purpose/use of AI | What are the applications or uses of AI in predicting cardiac arrest |
| AI branches | The branches/areas used |
| AI models/ algorithms | The specific AI models or algorithms used |
| Platform | The platform in which the AI technology was implemented |
| Outcome | The outcome of the AI technology |
| Population Characteristics |  |
| Age Group | The age-group the participants belonged to |
| Health condition | Health status of participants |
| Dataset Characteristics |  |
| Data sources | Source of data used for the development and validation of AI algorithms |
| Data types | Type of data that were used for the development and validation of AI models/ algorithms |
| Dataset size | The total number of data that used for development and validation of AI models/ algorithms. |
| Type of validation | How the dataset was split/used to develop and test the proposed models/ algorithms |

: Data Extraction Form
