# Supplementary material for: Artificial Intelligence in Predicting Cardiac Arrest: Scoping Review
Source: JMIR Med Inform. 2021 Dec 17;9(12):e30798. doi: 10.2196/30798 (PMC8726033; doi:10.2196/30798)
Supplement: Multimedia Appendix 3 [file medinform_v9i12e30798_app3.docx]

Multimedia Appendix 3

: Characteristics of the included studies and features of AI techniques

| ID | Authors | Year | Country | Publication Type | AI branch | Specific type | Platform | Data type category | Data Source | Population | Datasize | Validation type | Outcome Measures |
| --- | --- | --- | --- | --- | --- | --- | --- | --- | --- | --- | --- | --- | --- |
| 1 | Park et. al. | 2021 | China | Research Article | DL | RNN | PC | Clinical data | Clinical Setting | Pediatrics | ≥10,000 | N/A | AUROC |
| 2 | Murugappan et. al. | 2021 | USA | Research Article | ML | SVM | PC | Clinical data | Public database | Adult | <1000 | 10-fold cross validation | Accuracy |
| 3 | L et. al. | 2021 | India | Research Article | ML & DL | DT, RF, Adaptive Boosting, NB, LR, KNN, XGB, GBT | PC | Demographic data, Clinical data | Public database | Adult | 1000-9999 | 5-fold cross validation | Accuracy, Precision, Recall, F-1-score |
| 4 | Jang et. al | 2020 | S. Korea | Research Article | DL | ANN, MLP, LSTM, hybrid | PC | Clinical data | Clinical Setting | Adult | ≥10,000 | Training-test split | AUROC |
| 5 | Rohila et. al., | 2020 | India | Research Article | ML | SVM and DT | PC | Clinical data | Public database | Adult | N/A | N/A | Accuracy, Sensitivity, Specificity. |
| 6 | Hong et. al. | 2020 | S. Korea | Research Article | ML & DL | LR, RF, RNN | PC | Demographics, Clinical data | Clinical Setting | Adult | ≥10,000 | Training-test split | AUROC, AUPRC |
| 7 | Elola et. al. | 2020 | Spain | Conference Proceeding | ML | RF | PC | Clinical data | Clinical Setting | Adult | <1000 | 5-Fold cross-validation | Sensitivity, Recall, specificity, precision, AUROC and AUPRC. |
| 8 | Ueno et. al. | 2020 | India | Research Article | ML | RF | PC | Clinical data, Laboratory data | Clinical Setting | Adult | ≥10,000 | Training-test split | AUC |
| 9 | Balachander et. al | 2020 | India | Research Article | ML | RF | PC | Demographic data, Clinical data, Laboratory data | Clinical Setting | Adult | <1000 | N/A | Accuracy |
| 10 | Fernandes et. al. | 2020 | Portugal | Research Article | ML | LR, RF, and Extreme gradient boosting | PC | Demographic data, Clinical data, Laboratory data | Clinical Setting | Adult | ≥10,000 | N/A | AUROC, precision-recall curves |
| 11 | Kumar et. al. | 2020 | India | Conference Proceeding | ML & DL | CNN and RF | PC | Demographic data, Clinical data, Laboratory data | Public database | Other | Not mentioned | 5-Fold cross-validation | Accuracy |
| 12 | Kwon et. al. | 2020 | S. Korea | Research Article | DL | DL based algorithm | PC | Clinical data | Clinical Setting | Adult | ≥10,000 | Internal and External | NPV, PPV, AUC |
| 13 | Cho et. al. | 2020 | S. Korea | Research Article | DL | RNN | PC | Clinical data | Clinical Setting | Adult | Not mentioned | External validation | AUROC, AUPRC |
| 14 | Chang et. al. | 2019 | Taiwan | Conference Proceeding | ML & DL | RF and LSTM | PC | Demographic data, Clinical data | Clinical Setting | Adult | 1000-9999 | leave-one-out cross-validation | AUPRC |
| 15 | Liu et. al. | 2019 | Taiwan | Conference Proceeding | ML & DL | AdaBoost, RF, NB, DT, CART, LR, LSTM, CNN | PC | Demographic data, Clinical Data | Clinical Setting | Adult | ≥10,000 | 3-fold cross-validation | AUROC, AUPRC, f-1 |
| 16 | Layeghian et. al. | 2019 | Iran | Research Article | ML & DL | SVM, DT, LR, KNN, GaussianNB, Gradient Boosting, XGBoost, RF | PC | Demographic data, Clinical data | Public database | Adult | 1000-9999 | 10-fold cross-validation | Accuracy, Precision, sensitivity, F1-score, AUC, FPR - precision more important than sensitivity |
| 17 | Majumder et. al. | 2019 | USA | Research Article | ML | DT | Wearable | Clinical data | Other | Adult | <1000 | Train-test split | Accuracy |
| 18 | Shashikant et. al | 2019 | India | Research Article | ML | LR, DT, RF | PC | Clinical data | Public database | Adult | 1000-9999 | 10-fold cross-validation | Accuracy, precision, sensitivity, specificity, F1 score, and AUC |
| 19 | Chauhan et. al. | 2019 | India | Conference Proceeding | ML & DL | SVM, RF, DT, LR, ANN | PC | Demographic data, Clinical data, laboratory data | Other | Adult | Not mentioned | N/A | Accuracy |
| 20 | Lai et. al. | 2019 | China | Research Article | ML & DL | KNN, DT, NB, RF, SVM | PC | Demographic data, Clinical data | Public database | Adult | <1000 | 5-fold cross-validation | Sensitivity, Specificity, Accuracy |
| 21 | Kim et. al. | 2019 | S. Korea | Research Article | DL | LSTM | PC | Clinical data | Clinical Setting | Adult | ≥10,000 | External Validation | AUROC, Sensitivity, Specificity, positive predictive value, accuracy |
| 22 | Karankar et. al. | 2018 | India | Conference Proceeding | ML | NB | PC | Clinical data | Public database | Other | <1000 | Training - Test split | Accuracy |
| 23 | Akrivos et. al. | 2018 | Greece | Research Article | ML | Multichannel Hidden Markov Model | PC | Clinical data | Public database | Adult | <1000 | 8-fold cross-validation | Sensitivity |
| 24 | Alfarhan et. al. | 2018 | Malaysia | Research Article | DL | KNN | PC | Clinical data | Public database | Adult | <1000 | Ten-fold cross-validation | Accuracy |
| 25 | Amezquita et. al. | 2018 | USA | Research Article | DL | EPNN | PC | Demographic data, Clinical data | Clinical Setting | Adult | Not mentioned | Train-test split | Accuracy and Prediction time. |
| 26 | Kwon et. al. | 2018 | S. Korea | Research Article | DL | RNN | PC | Clinical data | Clinical Setting | Adult | ≥10,000 | External Validation | AUROC, AUPRC, # of false alarms, Sensitivity |
| 27 | Raka et. al. | 2017 | Australia | Research Article | ML | LDA, SVM | PC | Clinical data | Public database | Adult | <1000 | 5-fold cross-validation | Accuracy |
| 28 | Tapas et. al. | 2017 | India | Research Article | ML & DL | LogiBoost, RF, MLP | PC | Clinical data, Biological data | Public database | Adult | ≥10,000 | Train-test split | Accuracy and Precision |
| 29 | Tylman et. al. | 2016 | Poland | Research Article | ML | Bayesian networks | PC | Clinical data, Laboratory data | Clinical Setting | Adult | <1000 | External validation | Sensitivity |
| 30 | Somanchi et. al. | 2015 | USA | Conference Proceeding | ML | SVM And LR | PC | Demographic data, Clinical data, Laboratory data | Clinical Setting | Adult | ≥10,000 | 5-Fold cross-validation | Recall, False Positive rate from prediction, AUC |
| 31 | Kennedy et. al. | 2015 | USA | Research Article | ML | SVM | PC | Clinical data | Clinical Setting | Pediatrics | <1000 | 10-fold cross-validation | Accuracy, AUROC. |
| 32 | Churpek et. al. | 2015 | USA | Research Article | ML & DL | LR, Tree-based, KNN, SVM, Neural Networks | PC | Demographic data, Clinical data, Laboratory data | Clinical Setting | Adult | ≥10,000 | 10-fold cross-validation | AUC, sensitivity |
| 33 | Murugappan et. al. | 2015 | Malaysia | Research Article | ML & DL | KNN and Fuzzy classifier | PC | Clinical data | Public database | Adult | <1000 | 10-fold cross-validation | Accuracy, Sensitivity, specificity. |
| 34 | Rajendra et. al. | 2015 | Malaysia | Research Article | ML & DL | kNN, DT, SVM, and PNN | PC | Clinical data | Public database | Adult | <1000 | 10-fold cross-validation | Accuracy and Sensitivity and Specificity |
| 35 | Goldstein et. al. | 2014 | USA | Research Article | ML | RF | PC | Demographic data, Clinical data | Public database | Adult | ≥10,000 | Train-test split | Concordance statistic |
| 36 | Ebrahimzadeh et. al | 2014 | Iran | Research Article | DL | KNN, MLP | PC | Demographic data, Clinical data | Public database | Adult | <1000 | leave-one-out cross-validation | Accuracy, Sensitivity, Specificity, and Precision |
| 37 | Liu et. al. | 2014 | Singa-pore | Research Article | ML | RF | PC | Clinical data | Clinical Setting | Adult | <1000 | 10-fold cross-validation | AUROC, Sensitivity, and Specificity |
| 38 | Murukesan et. al. | 2014 | Malaysia | Research Article | ML & DL | Sequential Feature Selection, SVM, PNN | Wearable | Clinical data | Public database | Adult | <1000 | 10-fold cross-validation | Prediction rate |
| 39 | Collins et. al. | 2013 | USA | Research Article | ML | N/A | PC | Clinical data | Clinical Setting | Adult | ≥10,000 | N/A | Mean difference, t-test, risk-stratified sample |
| 40 | Badriyah et. al. | 2013 | UK | Research Article | ML | DT | PC | Clinica data | Clinical Setting | Adult | ≥10,000 | Train-test split | AUROC |
| 41 | Rohollah et. al. | 2020 | Iran | Conference Proceeding | ML | Tree-bagger and SVM | PC | Clinical data | Clinical Setting | Adult | <1000 | 10-fold cross-validation | Accuracy, sensitivity, specificity, precision |
| 42 | Fujita et. al. | 2016 | USA | Research Article | ML & DL | DT, kNN, and SVM | PC | Clinical data | Public database | Adult | <1000 | 10-fold cross-validation | Accuracy, sensitivity, and specificity |
| 43 | Houshyarifar et. al. | 2016 | Iran | Research Article | ML & DL | kNN and SVM | PC | Clinical data | Public database | Adult | <1000 | 10-fold cross validation | Accuracy, sensitivity, and specificity |
| 44 | Ting et. al. | 2021 | China | Research Article | ML | XGBoost | PC | Clinical data, radiology data, laboratory data | Clinical Setting | Adult | <1000 | Train-test split | AUROC, accuracy, sensitivity |
| 45 | Nakajima et. al. | 2020 | Japan | Research Article | ML & DL | LR, RF, GBT, SVM, NB, NN | PC | Clinical data, demographic, radiology | Clinical Setting | Adult | <1000 | 4-fold cross-validation | Specificity, AUC, sensitivity |
| 46 | Ebrahimzadeh et. al | 2019 | Iran | Research Article | ML & DL | MLP, kNN, SVM, ME | PC | Clinical data | Public database | Adult | <1000 | External Validation | Sensitivity, specificity, and accuracy |
| 47 | Joyce et. al. | 2017 | S. Korea | Conference Proceeding | ML | LR, Transfer learning | PC | Clinical data | Public database | Adult | <1000 | Train-test split | Accuracy, AUC |
